# Uppsala Consensus Statement on Environmental Contaminants and the Global Obesity Epidemic

**DOI:** 10.1289/ehp.1511115

**Published:** 2016-05-01

**Authors:** Lars Lind, P. Monica Lind, Margareta H. Lejonklou, Linda Dunder, Åke Bergman, Carlos Guerrero-Bosagna, Erik Lampa, Hong Kyu Lee, Juliette Legler, Angel Nadal, Youngmi Kim Pak, Richard P. Phipps, Laura N. Vandenberg, Daniel Zalko, Marlene Ågerstrand, Mattias Öberg, Bruce Blumberg, Jerrold J. Heindel, Linda S. Birnbaum

**Affiliations:** 1 Cardiovascular Epidemiology, Department of Medical Sciences, Uppsala University, Uppsala, Sweden; 2 Department of Occupational and Environmental Medicine, Uppsala University, Uppsala, Sweden; 3 Swedish Toxicology Sciences Research Center (Swetox), Södertalje, Sweden; 4 Department of Physics, Chemistry and Biology, Linköping University, Linköping, Sweden; 5 Uppsala Clinical Research (UCR) Center, Uppsala, Sweden; 6 Department of Internal Medicine, College of Medicine, Eulji University, Seoul, South Korea; 7 Institute for Environmental Studies, Vrije Universiteit Amsterdam, Amsterdam, Netherlands; 8 CIBER de Diabetes y Enfermedades Metabólicas Asociadas, CIBERDEM Instituto de Bioingeniería, Universidad Miguel Hernández de Elche, Elche, Alicante, Spain; 9 Department of Physiology, College of Medicine, Kyung Hee University, Seoul, Korea; 10 Department of Environmental Medicine, University of Rochester, Rochester, New York, USA; 11 Department of Environmental Health Sciences, School of Public Health and Health Sciences, University of Massachusetts Amherst, Amherst, Massachusetts, USA; 12 Institut National de la Recherche Agronomique (INRA) UMR1331 (Unité Mixe de Recherche 1331), Toxalim (Research Centre in Food Toxicology), Toulouse, France; 13 University of Toulouse, INPT (Institut National Polytechnique de Toulouse), UPS (Universite Paul Sabatier), Toulouse, France; 14 Department of Environmental Science and Analytic Chemistry, Stockholm University, Stockholm, Sweden; 15 Department of Developmental and Cell Biology, University of California, Irvine, Irvine, California, USA; 16 National Institute of Environmental Health Sciences, National Institutes of Health, Department of Health and Human Services, Research Triangle Park, USA

## Abstract

Summary: From the lectures presented at the 2nd International Workshop on Obesity and Environmental Contaminants, which was held in Uppsala, Sweden, on 8–9 October 2015, it became evident that the findings from numerous animal and epidemiological studies are consistent with the hypothesis that environmental contaminants could contribute to the global obesity epidemic. To increase awareness of this important issue among scientists, regulatory agencies, politicians, chemical industry management, and the general public, the authors summarize compelling scientific evidence that supports the hypothesis and discuss actions that could restrict the possible harmful effects of environmental contaminants on obesity.

## Introduction

Obesity is a growing problem, not only in developed countries but also in the developing world. Because obesity is associated with cardiovascular disease, diabetes, reproductive disorders, and some common cancers, the global obesity epidemic is contributing to a major negative impact on human health. It has been estimated that the cost of medical care attributable to obesity in the United States exceeds $200 billion USD a year (Cawley and Meyerhoefer 2012).

The prevailing view among the general public and many physicians is that the massive increase in obesity observed over the previous three decades (Ng et al. 2014; Ogden et al. 2014) is caused by bad habits that include an increase in food intake and a reduction in exercise. This generally adopted view proposes that the balance of calorie consumption and exercise alone are sufficient to explain the global obesity epidemic.

## Workshop on Obesity and Environmental Contaminants

On 8–9 October 2015, several leading researchers met for the 2nd International Workshop on Obesity and Environmental Contaminants in Uppsala, Sweden. From the lectures presented at the workshop, it became evident that the findings from numerous animal and epidemiological studies are consistent with the hypothesis that environmental pollutants could contribute to the global obesity epidemic. In this article, the authors present some of these findings to inform other scientists, regulatory agencies, politicians, industrial management, and the interested public about the important role that environmental contaminants could be playing in the burgeoning obesity epidemic. They also suggest actions that can be taken now to reduce and prevent the effects of environmental contaminants on obesity.

## Obesogen Hypothesis

Growing scientific evidence indicates that the imbalance of caloric consumption and exercise does not fully explain the obesity epidemic. Nor can DNA mutations explain the global obesity trends, because changes in genes rarely occur over the short period of human history during which obesity has become a problem. A recently published study that used data from the U.S. National Health and Nutrition Examination Survey (NHANES), which has been collected over nearly four decades, showed an increase in both caloric intake and body mass index (BMI) over time (Brown et al. 2015). Surprisingly, this study demonstrated that for a given amount of caloric intake, macronutrient intake, or leisure-time physical activity, the predicted BMI was significantly higher in 2006 than in 1998. In their article, Brown et al. (2015) concluded that “Factors other than diet and physical activity may be contributing to the increase in BMI over time.”

Over the past decade, many research studies have evaluated the hypothesis that environmental contaminants could contribute to obesity, and evidence is rapidly accumulating in support of this hypothesis (Gore et al. 2015; Grun and Blumberg 2009). Of special interest are studies that have evaluated prenatal exposures because exposure during this sensitive period of early development is likely to induce more profound or irreversible effects than are exposures that occur later in life.

Experimental animals exposed to environmental contaminants such as bisphenol A (BPA), dichlorodiphenyltrichloroethane (DDT), phthalates, perfluorooctanoic acid (PFOA), dioxins, and tributyltin (TBT) during pregnancy are more likely to give birth to offspring that display increased fat accumulation leading to obesity (Angle et al. 2013; Grun et al. 2006; Hao et al. 2013; Hines et al. 2009; Manikkam et al. 2013; Skinner et al. 2013; Somm et al. 2009; van Esterik et al. 2015). The exposure levels used in many of these studies were similar to those measured in human populations. La Merrill et al. (2014) showed that exposure to DDT during pregnancy can reduce basal metabolism in the offspring, a fact that could explain why these offspring gain extra weight for a given energy intake. Furthermore, exposure to BPA has been reported to lead to increased food intake due to changes in the brain resulting in stimulated appetite (Mackay et al. 2013).

During the workshop, attendees also discussed human studies demonstrating that exposure of pregnant women to environmental contaminants is associated with increased weight gain of their babies. Numerous studies have shown that prenatal exposure to dichlorodiphenyldichloroethylene (DDE), a DDT metabolite, is associated with rapid weight gain in children (Iszatt et al. 2015; Valvi et al. 2014) and that higher levels of DDE in the blood of pregnant mothers is associated with obesity in the adult offspring (Karmaus et al. 2009). Similar associations have been seen with other pollutants such as hexachlorobenzene (HCB) (Smink et al. 2008; Valvi et al. 2014), as well as mixtures of organochlorines (Agay-Shay et al. 2015). The findings from these studies support the hypothesis that the obesogenic effects noted in experimental animals are also relevant for humans.

## Obesogens and Metabolic Disruption

To highlight the importance of obesogens and metabolic disruption, the workshop attendees also discussed the multiple health effects of obesity. For example, when fat accumulates in abnormal locations, such as in the liver or surrounding the gut, heart, and kidneys, multiple other disorders often develop such as lipid disturbances, fatty liver, diabetes, and high blood pressure. Obesity and these other accompanying disorders are major risk factors for additional diseases that occur later in life such as cardiovascular disease, some common cancers, reproductive disorders, and even dementia, which are all responsible for reduced quality of life and premature death. Not only have environmental contaminants been linked to the development of obesity, but several pollutants, such as polychlorinated biphenyls (PCBs), dioxins, BPA, and pesticides have also been associated with mitochondrial dysfunction, lipid disturbances, insulin resistance, diabetes, and high blood pressure in both animal and human studies (La Merrill et al. 2013; Lim et al. 2009). Moreover, these conditions can be induced experimentally in mice by environmental contaminants independently of obesity (Alonso-Magdalena et al. 2011; Gore et al. 2015). Thus, animal studies indicate that exposure to environmental contaminants may be a contributing factor not only to obesity but also to diseases associated with obesity and an altered metabolism.

## Impact on Future Generations

A recent and important finding in mice showed that the obesogenic effect of TBT appears not only in the first generation of mice but is passed on to further generations (Chamorro-Garcia et al. 2013). This phenomenon, called transgenerational effect, has been shown to be due to epigenetic mechanisms in other experiments. Epigenetic events do not involve alterations in the genetic code, but rather mitotically stable changes in the regulation of gene expression (Skinner et al. 2013). In the case of TBT, this contaminant activates a part of the cellular machinery that continues to drive increased development of fat cells in future generations. Such effects are more pronounced when the experimental animals are maintained on a high-fat diet (Chamorro-Garcia et al. 2013).

## Recommendations for an Action Plan

Based on results discussed at the workshop, the authors suggested several actions that should be taken to restrict the potentially harmful effects of environmental contaminants on metabolism:

Increase research initiatives and funding to further explore mechanisms associated with chemical obesogen-induced metabolic disruptions, to examine mixtures, and to use exposure levels relevant to those encountered by human populations.
Educate physicians and other health care professionals regarding the effects of environmental contaminants on metabolism to increase the awareness of this problem, and how they could guide their patients, as well as the general population, to limit their exposure to these contaminants.
Ensure that knowledge of obesogenic environmental chemicals is incorporated into regulatory and policy making.
Demand that new chemicals that are to be released onto the market are tested in an appropriate fashion regarding their effects on metabolism.
Demand that all chemicals included in consumer products are disclosed in order to increase public awareness of their use and to provide individuals with the information they need to avoid exposures.
Find additional ways to increase public awareness about factors beyond caloric balance that are involved in obesity development, including the role of some environmental contaminants.
Increase awareness about the potential of these exposures to generate effects in future generations. This action item should also include education on how to avoid exposure to these contaminants.

## Summary and Conclusions

In conclusion, since there are now numerous animal and epidemiological studies indicating that environmental pollutants could contribute to the global obesity epidemic, there is an urgent need to reduce the burden of environmental contaminants so that obesity does not become the normal outlook in the future. The workshop attendees concluded that public health efforts should focus on the importance of early obesity prevention by means of reducing chemical exposures, rather than only treating the established disease. Just as a bad start can last a lifetime and beyond, a good start can last a lifetime as well.
